# Supplementary material for: Heat adaptation of phage T7 under an extended genetic code
Source: Virus Evol. 2021 Dec 1;7(2):veab100. doi: 10.1093/ve/veab100 (PMC8923235; doi:10.1093/ve/veab100)
Supplement: veab100_Supp [file veab100_supp.zip › Sequencing_Analysis.zip!/Sequencing_Analysis/A1/evidence/RA_33.html]

BRESEQ :: Evidence


| Read alignment evidence... | | | | | | | | | | | |
| --- | --- | --- | --- | --- | --- | --- | --- | --- | --- | --- | --- |
|  | seq id | position | | ref | new | freq | score (cons/poly) | reads | annotation | genes | product |
| \* | NC\_001604.1 | 28,493 | 0 | A | G | 44.5% | 449.7 / inf | 2782 | intergenic (–/–) | *–/–* | –/– |
| Reads supporting (aligned to +/- strand):  **ref** base A (702/829);  **new** base G (560/665);  **total** (1275/1507) | | | | | | | | | | | |
| Fisher's exact test for biased strand distribution *p*-value = 9.69e-01 | | | | | | | | | | | |
| Kolmogorov-Smirnov test that lower quality scores support variant *p*-value = 4.34e-01 | | | | | | | | | | | |

Only 100 of 2833 total aligned reads displayed.  

`GTTCTGGACCCACTGGCTATCATGGGGTCTTCCGCTGCGAGTGCTTACGCATCCGGTGCGTTCGACTCTAAGTCCACAACTAAGGCACCTATTGTTGCCGCTAAAGGAACCAAGACGGGGAGGTAATGAGCTATGAGTAAAATTGAATCTGCCCTTCAAGCGGCACAACCGGGACTCTCTCGGTTACGTGGTGGTGCTGGAGGTATGGGCTATCGTGCAGCAACCACTCAGGCCGAACAGCCAAGGTCAAGCCTATTGGACACCATTGGTCGGTTCGCTAAGGCTGGTGCCGATATGTATACCGCTAAGGAACAACGAGCACGAGACCTAGCTGATGAACGCTCTAACGAGATTATCCGTAAGCTGACCCCTGA‑GCAACGTCGAGAAGCTCTCAACAACGGGACCCTTCTGTATCAGGATGACCCATACGCTATGGAAGCACTCCGAGTCAAGACTGGTCGTAACGCTGCGTATCTTGTGGACGATGACGTTATGCAGAAGATAAAAGAGGGTGTCTTCCGTACTCGCGAAGAGATGGAAGAGTATCGCCATAGTCGCCTTCAAGAGGGCGCTAAGGTATACGCTGAGCAGTTCGGCATCG  >  NC_001604.1/28193‑28793`  

                                                                                                                                                                                                                                                                                                            `|`                                                                                                                                                                                                                                                                                                               
    `gggACCCACTGGCTATCATGGGGTCTTCCGCTGCGAGTGCTTACGCATCCGGTGCGTTCGACTCTAAGTCCACAACTAAGGCACCTATTGTTGCCGCTAAAGGAACCAAGACGGGGAGGTAATGAGCTATGAGTAAAATTGAATCTGCCCTTCAAGCGGCACAACCGGGACTCTCTCGGTTACGTGGTGGTGCTGGAGGTATGGGCTATCGTGCAGCAACCACTCAGGCCGAACAGCCAAGGTCAAGCCTATTGGACACCATTGGTCGGTTCGCTAAGGCTGGTGCCGATATGTATACCGc                                                                                                                                                                                                                                                                                                           <  1:329637/300‑1 (MQ=255)`  
        `cccACTGGCTATCATGGGGTCTTCCGCTGCGAGTGCTTACGCATCCGGTGCGTTCGACTCTAAGTCCACAACTAAGGCACCTATTGTTGCCGCTAAAGGAACCAAGACGGGGAGGTAATGAGCTATGAGTAAAATTGAATCTGCCCTTCAAGCGGCACAACCGGGACTCTCTCGGTTACGTGGTGGTGCTGGAGGTATGGGCTATCGTGCAGCAACCACTCAGGCCTAACAGCCAAGGTCAAGCCTATTGGACACCATTGGTCGGTTCGCTAAGGCTGGTGCCGCTATGTATACCGCTAAg                                                                                                                                                                                                                                                                                                       >  1:142617/1‑301 (MQ=255)`  
         `cacttGGCTATCATGGGGTCTTCCGCTGCGAGTGCTTACGCATCCGGTGCGTTCGACTCTAAGTCCACAACTAAGGCACCTATTGTGGCCGCTAAAGGAACCAAGACGGGGAGGTAATGAGCTATGAGTAAAATTGAATCTGCCCTTCAAGCGGCACAACCGTGACTCTCTCGGTTACGTGGTGGTGCTGGAGGTATGGGCTATCGTGCAGCAACCACTCAGTCCGAACAGCCAAGGTCAAGCCTATTGGACACCATTGGTCGGTTCGCTAAGGATGGTGCCGATATGTATACCGCTAAgg                                                                                                                                                                                                                                                                                                      <  1:365944/297‑1 (MQ=255)`  
            `tgggtataagagacagcTTCCGTTGAGAGTTCCTACGCATCCGGTGCGTTCGACTTTAAGTCCACAACTAAGGCACCTATTGTTGCCGCTAAAGGAACCAAGACGGGGAGGTAATGAGCTATGAGTAAAATTGAATCTGCCCTTCAAGCGGCACAACCGGGACTCTCTCGGTTTCGTTGTGGTGCTTGAGGTATGGGCTATCGTGCAGCAACCACTCAGGCCGAACAGCCAAGGTCAAGCCTATTGGACACCATTGGTCGGTTCGCTAAGGCTTGTGCCGATATGTATACCGCTAAGGaac                                                                                                                                                                                                                                                                                                   <  1:64244/285‑1 (MQ=255)`  
            `cTGGCTATCATGGGGTCTTCCGCTGCGAGTGCTTACGCATCCGGTGCGTTAGACTCTAAGTCCACAACTAAGGCACCTATTGTTGCCGCTAAAGGAACCAAGACGGGGAGGTAATGAGCTATGAGTAAAATTGAATCTGCCCTTCAAGCGGCACAACCGGGACTCTATCGGTTACGTGGTGGTGCTGGAGGTAGGGGCTATGGGACGGAAACAACTAAGGCGGAACACCCAAGGTCAGGCCAATTGGAAACAATGGGTGGGTTGGCTAAGGCGGGTGAGGAGAGGTATGCGGCTACGGaca                                                                                                                                                                                                                                                                                                   >  1:200985/1‑299 (MQ=255)`  
                  `aTCATGGGGTCTTCCGCTGCGAGTGCTTACGCATCCGGTGCGTTCGACTCTAAGTCCACAACTAAGGCACCTATTGTTGCCGCTAAAGGAACCAAGACGGGGAGGTAATGAGCTATGAGTAAAATTGAATCTGCTCTTCAAGCGGCACAACCGGGACTCTCTCGGTTACGTGGTGGTGCTGGAGGTATGGGCTATCGTGCAGCAACCACTCAGGCCGAACAGCCAAGGTCAAGCCTATTGGACACCATTGGTCGGTTCGCTAAGGCTGGTGCCGATATGTATACCGCTAAGGaacctgtct                                                                                                                                                                                                                                                                                             >  1:325364/1‑295 (MQ=255)`  
                  `aTCATGGGGTCTTCCGCTGCGAGTGCTTACGCATCCGGTGCGTTCGACTCTAAGTCCACAACTAAGGCACCTATTGTTGCCGCTAAAGGAACCAAGACGGGGAGGTAATGAGCTATGAGTAAAATTGAATCTGCCCTTCAAGCGGCACAACCGGGACTCTCTCGGTTACGTGGTGGTGCTGGAGGTATGGGCTATCGTGCAGCAACCACGCAGGCCGAACAGCCAAGGTCAAGCCTATTGGACACCATTGGTCGGTTCGCTCAGCCTGGTGCCGATATGTATACCGCTAAGGAACAACGAg                                                                                                                                                                                                                                                                                             >  1:283159/1‑301 (MQ=255)`  
                           `tCTTCCGCTGCGAGTGCTTACGCATCCGGTGCGTTCGACTCTAAGTCCACAACTAAGGCACCTATTGTTGCCGCTAAAGGAACCAAGACGGGGAGGTAATGAGCTATGAGTAAAATTGAATCTGCCCTTCAAGCGGCACAACCGGGACTCTCTCGGTTACGTGGTGGTGCTGGAGGTATGGGCTATCGTGCAGCAACCACTCAGGCCGAACAGCCAAGGTCAAGCCTATTGGACACCATTGGTCGGTTCGCTAAGGCTGGTGCCGATATGTATACCGCTAAGGAACAACGAGCACGAGAcc                                                                                                                                                                                                                                                                                    <  1:1385/301‑1 (MQ=255)`  
                            `cTTCCGCTGCGAGTGCTTACGCATCCGGTGCGTTCGACTCTAAGTCCACAACTAAGGCACCTATTGTTGCCGCTAAAGGAACCAAGACTGGGAGGTAATGAGCTATGAGTAAAATTGAATCTGCCCTTCAAGCGGCACAACCGGGACTCTCTCGGTTACGTGGTGGTGCTGGAGGTATGGGCTATCGTGCAGCAACCACTCAGGCCGAACAGCCAAGGTCAAGCCTATTTGACACCATTGGTCGGTTCGCTAAGGCTGGTGCCGATATGTATACCGCTAAGGAACAACGAGCACGAGAccc                                                                                                                                                                                                                                                                                   >  1:147634/1‑300 (MQ=255)`  
                               `ccGCTGCGAGTGCTTACGCATCCGGTGCGTTCGACTCTAAGTCCACAACTAAGGCACCTATTGTTGCCGCTAAAGGAACCAAGACGGGGAGGTAATGAGCTATGAGTAAAATTGAATCTGCCCTTCAAGCGTCACAACCGGGACTCTCTCGGTTACGTGGTGGTGCTGGAGGTATGGGCTATCGTGCAGCAACCACTCAGGCCGAACAGCCAAGGTCAAGCCTATTGGACACCCTTGGTCGGTTCGCTAAGGCTGGTGCCGATATGTATACCGCTACGGAACAACGAGCACGAGACCTAGc                                                                                                                                                                                                                                                                                >  1:394141/1‑301 (MQ=255)`  
                                                `ccctCCGGTGCTTTCCACTCTAAGTCCACAACTACGGGCCCTATTTTTGCCGCTAACGGAACCAAGACGGGGAGGTAATGGGCTATGAGTAAAATTGAATCTGCCCTTCAAGCGGCACAACCGGGACTCTCTCGGTTACGTGGTGGTGCTGGAGGTATGGGCTATCGTGCAGCAACCACTCAGGCCGAACAGCCAAGGTCAAGCCTATTGGACACCATTGGTCGGTTCGATAAGGCTGGTGCCGATATGTATACCGCTAAGGAACAACGAGCACGAGACCTAGCTGATGAACGCTCTAACg                                                                                                                                                                                                                                                               <  1:258120/298‑1 (MQ=255)`  
                                                 `cATCCGGTGCGTTCGACTCTAAGTCCACAACTAAGGCACCTATTGTTTCCGCTAAAGGAACCAAGACGGGGAGGTACTGAGCTATGAGTAAAATTGAATATGCCCTTCAAGCGGCACAACCGGGACTCTCTCGGTTACGTGGTGGTGCTGGAGGTATGGGCTATCGTGTAGCAACCACTCAGGCCGAACAGCCAAGGTCAAGCCTATTGGACACCATTGGTCGGTTCGCTAAGGCTGGTGCCGATATGGATGCCGCTAAGGAACAACAAGCACGCGAACTCGCTGATGAACGCTCTAACga                                                                                                                                                                                                                                                              >  1:246659/1‑301 (MQ=255)`  
                                                 `cATCCGGTGCGTTCGACTCTAAGTCCACAACTAAGGCACCTATTGTTGCCGCTAAAGGAACCAAGACGGGGAGGTAATGAGCTATGAGTAAAATTGAATCTGCCCTTCAAGCGGCACAACCGGGACTCTCTCGGTTACGTGGTGGTGCTGGAGGTATGGGCTATCGTGCAGCAACCACTCAGGCCGAACAGCCAAGGTCAAGCCTATTGGACACCATTGGTCGGTTCGCTAAGGCTGGTGCCGATATGTATGCCGCTAAGGAACAACGAGCACGAGACCTAGCTGATGAACGCTCTAACga                                                                                                                                                                                                                                                              >  1:388776/1‑301 (MQ=255)`  
                                                         `gCGTTCGACTCTAAGTCCACAACTAAGGCACCTATTGTTGCCGCTAAAGGAACCAAGACGGGGAGGTAATGAGCTATGAGTAAAATTGAATCTGCCCTTCAAGCGGCACAACCGGGACTCTCTCGGTTACGTGGTGATGCTGGAGGTATGGGCTATCGTGCAGCAACCACTCAGGCCGAACAGCCAAGGTCAAGCCTATTGGACACCATTGGTCGGTTCGCTAAGGCTGGTGCCGATATGTATGCCGCTAAGGAACAACAAGCACGAACCCTAGCTGATGAACGATCTAACGAGATTATcc                                                                                                                                                                                                                                                      >  1:170784/1‑301 (MQ=255)`  
                                                           `gTTCGACTCTAAGTCCACAACTAAGGCACCTATTGTTGCCGCTAAAGGAACCAAGACGGGGAGGTAATGAGCTATGAGTAAAATTAAATCTGCCCTTCAAGCGGCACAACCGGGACTCTCTCGGTTACGTGGTGGTGCTGGAGGTATGGGCTAGCGTGCAGCAACCACTCAGGCCGAACAGCCAAGTTCAAGCCTATTGGACACCATTGGTCGGTTCGCTAAGGCTGGTGCCGATACGTATACCGCTTAGGAACCACGAGCACGAGACCTAGCTGATTAACGCTCTAACGAGATTATCCGt                                                                                                                                                                                                                                                    >  1:35964/1‑301 (MQ=255)`  
                                                               `gACTCTAAGTCCACAACTAAGGCACCTATTGTTGCCGATAAAGGAACCAAGACGGGGGGGTAATGAGCTATGAGTAAAATTGAATCTGCCCTTCAAGCGGCACAACCGGGACTCTCTCGGTTACGTGGTGGTGCTGGAGGTATGGGCTATCGTGCAGCAACCACTCAGGCCGAACAGCCAAGGTCAAGCCTATTGGACACCATTGGTCGGTTCGCTAAGGCTGGTGCCGATATGTATACCGCTAAGGAACAACGAGCACGAGACCTAGCTGATGAACGCTCTAACGAGATTATCCATAAGc                                                                                                                                                                                                                                                <  1:476873/301‑1 (MQ=255)`  
                                                               `gACTCTAAGTAAACAACTAAGGCACCTATTGGTGCCGCGAAAGGAACCAAGACGGGGAGGTAATGAGCTATGAGTAAAATTGAATCTGCCCTTCAAGCGGCACAACCGGGACTCTCTCGGTTACGTTGTGGTGCTGGAGGTATGGGCTATCGTGCAGCAACCACTCAGGCCGAACAGCCAAGGTCAAGCATATTGGACACCATTGGTCGGTTCGCTAAGGCTGGTGCCGATATGTAGACCGCTAAGGAACAACGAGCACGAGACCTAGCTGATGAACGCTCTAACGAGATTATACGTAAGc                                                                                                                                                                                                                                                <  1:253801/301‑1 (MQ=255)`  
                                                               `agagacaggTCCACAACTAAGGCACCTATTGTTGCCGCCAAAGGAACCAAGACGGGGAGGTAATGAGCTATGAGTAAAATTGAATCTGCCCTTCAAGCGGTACAACCGGGACTCTCTCGGTTACGTGGTGGTGCTGGAGGTATGGGCTATCGTGCAGCAACCACTCAGGCCGAACAGCTAAGGTCAAGCCTATTGGACACCATTGGTCGGTTCGCTAAGGCTGGTGCCGATATGTATGCCGCTAAGGAACAACGAGCACGAGACCTAGCTGATGAACGCTCTAACGAGATTATCCGTAAGc                                                                                                                                                                                                                                                <  1:444064/293‑1 (MQ=255)`  
                                                               `aatgtgtataagagacagaaGGCACCGATTGTTGCCGCTAAAGGAACCAAGACGGGGAGGTAATGAGCTATGAGTAAAATTTAATCAGCCCTTCAAGCGGCACAACCTTGACTCTCTCGGTTACGTGGTGGTGCTGGATGTATGGGCTATCGTGCAGCAACCACTCAGGCCGAACAGCCAAGGTCAAGCCTATTGGACACCATTGGTCGGTTCGCTAAGGCTGGTGCCGATATGTATGCCGCTAAGGAACAACGAGCACGAGACCTAGCTGATGAACGCTCTAACGAGATTATCCGTAAGc                                                                                                                                                                                                                                                <  1:209756/283‑1 (MQ=255)`  
                                                                `aCTCTAAGTCCACAACTAAGGCACCTATTGTTGCCGCTAAAGGAACCAAGACGGGGAGGTAATGAGCTATGAGTAAAATTGAATCTGCCCTTCAAGCGGCACAACCGGGACTCTCTCGGTTACGTGGTGGTGATGGAGGTATGGGCTATCGTGCAGCAACCACTCAGGCCGAACAGCCAAGGTCAAGCCTATTGTACACCATTGGTCGGTTCGCTACGGCTGGTGCCGATATGTATACCGCTAAGGACCAACGAGCACGAGACCTAGCTGCTGAACGCTGTAACGAGATTATCCGTAAGCt                                                                                                                                                                                                                                               >  1:370557/1‑301 (MQ=255)`  
                                                                      `aGTCCACAACTAAGGCACCTATTGTTGCCGCTAAAGGAACCAAGACGGGGAGGTAATGAGCTATGAGTAAAATTGAATCTGCCCTTCAAGCGGCACAACAGGGACTCTCTCGGTTACGTGGTGGTGCTGGAGGTATGGGCTATCGTGCAGCAACCACTCAGGCCGAACAGCCAAGGTCAAGCCTATTGGACACCATTGGTCGGTTCGCTAAGGCTGGTGCCGATATGTATGCCGCTAAGGAACAACGAGCACGAGACCTAGCTGATGAACGCTCTAACGAGATTATCCGTAAGCTGAcacc                                                                                                                                                                                                                                         <  1:175851/301‑1 (MQ=255)`  
                                                                       `gTCCACAACTAAGGCACCTATTGTTGCCGCTAAAGGAACCAAGACGGGGAGGTAATGAGCTATGAGTAAAATTGAATCTGCCCTTCAAGCGGCACACCCGGGACTCTCTCGGTTAAGTTGTGGTGCTGGAGATATGGGCTATCGTGCAGCAACCACTCAGGCCGCACCGCCAATCTCATGCCTATTGGACACCATTGGTCGGTTCACTAAGGCTGGTGCCGATCTGTATACCGCTAAGGCACAACACGAACGAGCCCTAGCTGATGAACGCACTTACGAGATTATCCTTTAGCTGACCCCt                                                                                                                                                                                                                                        >  1:54518/1‑301 (MQ=255)`  
                                                                            `caACTAAGGCACCTATTGTTGCCGCTAAAGGAACCAAGACGGGGAGGTAATTCGCTATGAGTCACATTGAATCTGCCCTTCAAGCGGCACAACCGGTACTCTCTCGGTTACGTGGTGGTGCTGGAGGTATGGGCTATCGTGCAGCTACCACCCAGTCCGAACAGCCAAGGTCAAGCCTCTTGGACACCATTGGTCGTTTCGCTAAGGCTGTTGCCGATATGTATACCGCCAAGGAACAACGAGCACGAGACCTATCTGATGTACGCTCTAACGAGTTCATCCTTAAGCTTCCACCTGA‑GCa                                                                                                                                                                                                                                  >  1:46161/1‑301 (MQ=255)`  
                                                                            `caACTAAGGCACCTATTGTTGCCGCTAAAGGAACCAAGACGGGGAGGTAATGAGCTATGAGTAAAATTGAATCTGCCCTTCAAGCGGCACAACCGGGACTCTCTCTGTTACGTGGTGGTGCTGGAGGTATGGGCTATCGTGCAGCAACCACTCAGGCCGAACAGCCAAGTTCAATCCTATTGGACACCACTGGTCGGTTCGCTAAGCCTGGTGCCGATATGTATACCGCTCAGGAACAACGAGCACGAGACCTAGCTGATGAACGCTCTAACGAGATTATCCGTAATCTGACCCCTGT‑GCa                                                                                                                                                                                                                                  >  1:128010/1‑301 (MQ=255)`  
                                                                            `caACTAAGGCACCTATTGTTGCCGCTAAAGGAACCAAGACGGGGAGGTAATGAGCTATGAGTAAAATTGAATCTGCCCTTCAAGCGGCACAACCGGGACTCTCTCGGTTACGTGGTGGTGCTGGAGGTATGGGATATCGTGCAGCAACCACTCAGGCCGAACAGCCAAGGTCAAGCCTATTGGACACCATTGGTCGGTTCGCTAAGGCTGGTGCCGATATGTATACCGCTAAGGAACAACGAGCACGAGACCTAGCTGATGAACGCTCGAACGAGATTATCCGTAAGCTGACCCCTGA‑gaa                                                                                                                                                                                                                                  >  1:4095/1‑299 (MQ=255)`  
                                                                             `aaCTAAGGCACCTATTGTTGCCGCTAAAGGAACCAAGACGGGGAGGTAATGAGGTATGAGTAAATTTGAATCTGCCCTTCAAGCGGCACAACCGGGACTCTCTCGGTTACGTGGTGGTGCTGGAGGTATGGGCTATCGTGCAGCAACCACTCAGGCCGAACAGCCAAGGTCAAGCCTATTGGACACCATTGGTCGGTTCGCTAAGGCTGGTGCCGATATGTATACCGCTAAGGAACAACGAGCACGAGACCTAGCTGATGAACGCTCTAACGAGATTATCCGTAAGCTGACCCCTGA‑GCaa                                                                                                                                                                                                                                 <  1:429106/301‑1 (MQ=255)`  
                                                                              `aCTAAGGCACCTATTGTTGCCGCTAAAGGAACCAAGACGGGGAGGTAATTAGCTATGAGTAAAATTGAATCTGCCCTTCAAGCGGCACAACCGGGACTCTCTCGGTTACGTGGTGGTGCTGGAGGTATGGGCTATCGTGCAGCAACCACTCAGGCCGAAAAGCCAAGGTCAAGCCAATGCGACACCATTGGTCGGTTCGCTAAGGCTGGTGCCGATATGTCTGCCGCTAAGGAACAACGAGCACGAGACCTAGCTGATGAACGCTCTAACGCGATTATCCGTAAGCTGAACCCTGa‑acaac                                                                                                                                                                                                                                >  1:165628/1‑301 (MQ=255)`  
                                                                              `aCTAAGGCACCTATTGTTGCCGCTAAAGGAACCAAGACGGGGAGGTAATGAGCTATGAGTAAAATGGAATCTGCCCTTCAAGCGGCACAACCGGGACTCTCTCGGTTACGTGGTGGTGCTGGAGGTATGGGCTATCGTGCAGCAAACACTCAGGCCGCACAGCCAAGGTCAAGCCTATTGGACACCATTGGTCGGTTCGCTAAGGCTGGTGCCGACATGTATACCGATAAGGAACAACGAGCACGATACCTAGCTGCTGAACGCTCTAACGACATTATCCGTAACCTGACCCCTGC‑GCAAc                                                                                                                                                                                                                                >  1:123224/1‑301 (MQ=255)`  
                                                                               `cTAAGTCACCTATTGTTGCCGCTAAAGGAACCAAGACGGGGAGGTAATGAGCTATGAGTAAAATTGAATCTGCCCTTCAAGCGGCACAACCGGGACTCTCTCGGTTACGTGGTGGTGCTGGAGGTATGGGCTATCGTGCAGCAACCACTCAGGCCGAACAGCCAAGGTCAAGCCTATTTGCCACCATTTGTCGGTTCGCTAAGGCTGGTTCCGATCTGTATCCCCATAAGGAACAACGAGCACGAGACCTAGCTGATGCACGCTCTAACTAGATTATCCGTACGATGAccccgaagccaag                                                                                                                                                                                                                                >  1:290571/1‑292 (MQ=255)`  
                                                                                      `aCCTATTGTTGCCGCTAAAGGAACCAAGACGGGGAGGTAATGAGCTATGAGTAAAATTGAATCTGCCCTTCAAGCGGCACAACCGGGACTCTCTCGGTTACGTGGTGGTGCTGGAGGTATGGGCTATCGTGCAGCAACCACTCAGGCCGAACAGCCAAGGTCAAGCCTATTGGCCACCATTGGTCGGTTCGCTAAGGCTGGTGCCGATATGTATGCCGCTAAGGAACAACGAGCACGAGACCTAGCTGATGAACGCTCTAACGAGATTATCCGTAAGCTGACCCCTGC‑GCAACGTCgagca                                                                                                                                                                                                                        >  1:20548/1‑299 (MQ=255)`  
                                                                                       `gagatgtgtaaaagagacagAACCAAGACGGGGAGGTAATGAGCTATGAGTAAAATTGAATCTGCCCTTAAAGCGGCACAACCGGGACGCTCTCGGTTACGTGGTGGTGCTGGAGGTATGGGCTATCGTGCAGCAACCACTCAGGCCGAACAGCCAAGGTCAAGCCTATTGGACACCATTGGTCGGTTCGCTAAGGCTGGTGCCGATATGTATGCCGCTAAGGAACAACGAGCACGAGACCTAGCTGATGAACGCTCTAACGAGATTATCCGTAAGCTGACCCCTGA‑GCAACGTCGAGAAg                                                                                                                                                                                                                       <  1:43974/282‑1 (MQ=255)`  
                                                                                                                 `gtcGGGGAGTTAATGAGTTATGAGTAAAATTGAAGTTGCCCTTCAAGCTGCACAACCGGGACTCTCTCGGTTACGGGGTGGTGCTGGAGGTATGGCCTATCGTGCAGCAACCACTCAGGCCGAACAGCCAAGGTCAAGCCTATTGGACACCATTGGTCGGTTCGCTAAGGCTGGTGCCTATATGTATGCCGCTAAGGAACAACGAGCACGAGACCTAGCTGATGACCGCTCTAACGAGATTATCCGTAAGCTGACCCCTGA‑GCAACGTCGAGAAGCTCTCAACAACGGGACCCTTCTGTAt                                                                                                                                                                                             <  1:503563/299‑1 (MQ=255)`  
                                                                                                                 `gACTGGGAGGTAATGAGCTATTAGGAAAATGGAATCGGCCCTTCAAGCGGCACAACCGGGACTCTCTCGGTTACGTGGTGGTGCTGGAGGTATGGGCTATCGTGCAGCAACCACTCAGGCCGAACAGCCAAGGTCAAGCCTATTGGACACCATTGGTCGGTTCGCTAAGGCTGGTGCCGATATGTATGCCGCTAAGGAACAACGAGCACGAGACCTAGCTGATGAACGCTCTAACGAGATTATCCGTAAGCTGACCCCTGA‑GCAACGTCGAGAAGCTCTCAACAACGGGACCCTTCTGTAt                                                                                                                                                                                             <  1:448544/301‑1 (MQ=255)`  
                                                                                                                      `ggAGGTAATGAGCTATGAGTAAAATTGAATCTGCCCTTCAAGCGGCACAACCTGGACTCTCTCGGTTACGTGGTGGTGCTGGAGGTATGGGCTATCGTGCAGCAACCACTCAGGCCGAACAGCCAAGGTCAAGCCTATTGGACACCATTGGTCGGTTCGCTAAGGCTGGTGCCGATATGTATGCCGCTAAGGAACAACGAGCACGAGACCTAGCTGATGAACGCTCTAACGAGATTATCCGTAAGCTGACCCCTGA‑GCAACGTCGAGAAGCTCTCAACAACGGGACCCTTCTGTATCAGGa                                                                                                                                                                                        <  1:120153/301‑1 (MQ=255)`  
                                                                                                                         `ggTAATGAGCTATGAGTAAAATTGAATCTGCCCTTCAAGCGGCACAACCGGGACTCTCTCGGTTACGTGGTGGTGCTGGAGGTATGGGCTATCGTGCAGCAACCACTCAGGCCGAACAGCCAAGGTCAATCCTATTGGACACCATTGGTCGGTTCGCTAAGGCTGGTGCCGATATGTATACCGCTAAGGAACAACGAGCACGAGACCTAGCTGATGAACTCTCTAACGAGATTATCCGTAAGCTGACCCCTGA‑GCAACGTCGAGAAGCTCTCAACAACGGGACCCTTCTGTATCAGGATGa                                                                                                                                                                                     >  1:423383/1‑301 (MQ=255)`  
                                                                                                                         `ggTAATGAGCTATGAGTAAAATTGAATCTGCCCTTCAAGCGGCACAACCGGGACTCTCTCGGTTACGTGGTGGTGCTGGAGGTATGGGCTATCGTGCAGCAACCACTCAGGCCGAACAGCCAAGGTCAAGCCTATTGGACACCATTGGTCGGTTCGCTAAGGCTGGTGCCGATATGTATACCGCTAAGGAACAACGAGCACGAGACCTAGCTGATGAACGCTCTAACGAGATTATCCGTAAGCTGACCCCTGA‑GCAACGTCGAGAAGCTCTCAACAACGGGACCCTTcctgtctcttatac                                                                                                                                                                                     >  1:305641/1‑288 (MQ=255)`  
                                                                                                                            `aaataGCCATGACGAAAATTGAATCTGCCCTTCAAGCGGCACAACCGGGACTCTCTCGGTTACGTGGTGGTGCTGGAGGTATGGGCTATCGTGCAGCAACCACTCAGGCCGAACAGCCAAGGTCAAGCCTATTGGACACCATTGGTCGGTTCGCTAAGGCTGGTGCCGATATGTATGCCGCTAAGGAACAACGAGCACGAGACCTAGCTGATGAACGCTCTAACGAGATTATCCGTAAGCTGACCCCTGA‑GCAACGTCGAGAAGCTCTCAACAACGGGACCCTTCTGTATCAGGATGAccc                                                                                                                                                                                  <  1:401461/297‑1 (MQ=255)`  
                                                                                                                                `aGCTATGAGTAAAATTGAATCTGCCCTTCAAGCGGCACAACCGGGACTCTCTCGGTTACGTGGTGGTGCTGGAGGTATGGGCTATCGTGCAGCAACCACTCAGGCCGAACAGCCAAGGTCAAGCCTATTGGACACCATTGGTCGGTTCGCTAAGGCTGGTGCCGATATGTATACCGCTAAGGAACAACGAGCACGAGACCTAGCTGATGAACGCTCTAACGCGATTATTCGTAAGCTGACCCCTGA‑GCAACGTCGAGAAGCTCTCACCAAACGGAGCCTTCttgttctgtctcttatacac                                                                                                                                                                              >  1:68612/1‑282 (MQ=255)`  
                                                                                                                                `aGCTAAGAGTAAAATGGAATCTGCCCTTCAAGCGGCACAACCGGGACTCTCTCGGTTACGTGGTGGTGCTGGAGGTATGGGCTATCGTGCAGCAACCACTCAGGCCGAACAGCCAAGGTCAAGCCTATTGGACACCATTGGTCGGTTCGCTAAGGCTGGTGCCGATATGTATACCGCTAAGGAACAACGAGCACGAGACCTAGCTGATGAACGCTCTAACGAGATTATCCGTAAGCTGACCCCTGA‑GCAACGTCGAGAAGCTCTCAACAACGGGACCCTTCTGTATCAGGATGACCCATAc                                                                                                                                                                              <  1:265194/301‑1 (MQ=255)`  
                                                                                                                                  `taagagacagaaTTGAATCTGCCCTTCAAGCGGCACAACCGGGACTCTCTCGGTTACGTGGTGGTGCTGGAGGTATGGGATATCGTGCAGCAACCACTCAGGCCGAACAGCCAAGGTCAAGCCTATTGGACACCATTGGTCGGTTCGCTAAGGCTGGTGCCGATATGTATGCCGCTAAGGAACAACGAGCACGAGACCTAGCTGATGAACGCTCTAACGAGATTATCCGTAAGCTGACCCCTGA‑GCAACGTCGAGAAGCTCTCAACAACGGGACCCTTCTGTATCAGGATGACCCATACGc                                                                                                                                                                            <  1:384582/291‑1 (MQ=255)`  
                                                                                                                                      `gAGTAAAATTGAATCTGCCCTTCAAGCGGCACAACCGGGACTCTCTCGGTTACGTGGTGGTGCTGGAGGTATGGGCTATCGTGCAGCAACCACTCAGGCCGAACAGCCAAGGTCAAGCCTATTGGACACCATTGGTCGGTTCGCTAAGGCTGGTGCCGATATGTATACCGCTAAGGAACAACGAGCACGAGACCTAGCTGATGAACGCTCTAACGGGATTATCCGTAAGCTGACCCCTGA‑GCAACGTCGAGAAGCTCTCAACAACGGGACCCTTCTGTATCAGGATGACCCATACGCTATg                                                                                                                                                                        >  1:26935/1‑301 (MQ=255)`  
                                                                                                                                       `aGTAAAATTGAATCTGCCCTGCAAGCGGCACAACCGGGACTCTCTCGGTTACGTGGTGGTGCTGGAGGTATGGGCTATCGGGCAGCAACCACTCAGGCCGAACAGCCAAGGTCAAGCCTATTGGACACCATTGGTCGGTTCGCTAAGGCTGGTGCCGATATGTATACCGCTAAGGAACAACGAGCACGAGACCTAGCTGATGAACGCTCTAACGAGATTATCCGTAAGCTGACCCCTGA‑GCAACGTCGAGAAGCTCTCAACAACGGGACCCTTCTGTATCAGGATGACCCATACGCTATgg                                                                                                                                                                       <  1:86768/301‑1 (MQ=255)`  
                                                                                                                                          `aaaaTTGAATCTGCCCTTCAAGCGGCACAACCGGGACTCTCTCGGTTACGTGGTGGTGCTGGAGGTATGGGCTATCGTGCAGCAACCACTCAGGCCGAACAGCCAAGGTCAAGCCTATTGGACACCATTGGTCGGTTCGCTAAGGCTGGTGCCGATATGTATGCCGCTAAGGAACAACGAGCACGAGACCTAGCTGATGAACGATCTAACGAGATTATCCGTAAGCTGACCCCTGA‑GCAACGTCGAGAAGCTCTCAACAACGGGACCCTTCTGTATCAGGATGACCCATACGCTATGGAAg                                                                                                                                                                    <  1:490403/301‑1 (MQ=255)`  
                                                                                                                                           `aaaTTGAATCTGCCCTTCAAGCGGCACAACCGGGACTCTCTCGGTTACGTGGTGGTGCTGGAGGTATGGGCTATCGTGCAGCAACCACTCAGGCCGAACAGCCAAGGTCAAGCCTATTGGACACCATTGGTCGGTTCGCTAAGGCTGGTGCCGATATGTATACCGCTAAGGAACAACGAGCACGAGACCTAGCTGATGAACGCTCTAACGAGATTATCCGTAAGCTGACCCCTGA‑GCAACGTCGAGAAGCTCTCAACAACGGGACCCTTCTGTATCAGGATGACCCATACGCTATGGAAGc                                                                                                                                                                   <  1:180696/301‑1 (MQ=255)`  
                                                                                                                                            `aaTTGAATCTGCCCTTCAAGCGGCACAACCGGGACTCTCTCGGTTACGTGGTGGTGCTGGAGGTATGGGCTATCGTGCAGCAACCACTCAGGCCGAACAGCCAAGGTCAAGCCTATTGGACACCATTGGTCGGTTCGCTAAGGCTGGTGCCGATATGTATGCCGCTAAGGAACAACGAGCACGAGACCTAGCTGATGAACGCTCTAACGAGATTATCCGTAAGCTGACCCCTGA‑GCAACGTCGAGAAGCTCTCAACAACGGGACCCTTCTGTATCAGGATGACCCATACGCTCTGGAAGCa                                                                                                                                                                  >  1:105254/1‑301 (MQ=255)`  
                                                                                                                                                `gAATCTGCCCTTCAAGCGGCACAACCGGGACTCTCTCGGTTACGTGGTGGTGCTGGAGGTATGGGCTATCGTGCAGCAACCACTCAGGCCGAACAGCCAAGGTCAAGCCTATTGGACACCATTGGTCGGTTCGCTAAGGCTGGTGCCGATATGTATGCCGCTAAGGAACAACGAGCACGAGACCTAGCTGATGAACGCTCTAACGAGATTATCCGTAAGATGACCCCTGA‑GCAACGTCGAGAAGCTCTCAACAACGGGACCCTTCTGTATCAGGATGACTCATACGCTATGGAAGctgtcc                                                                                                                                                              >  1:83045/1‑296 (MQ=255)`  
                                                                                                                                                   `acTGCCCTTCCAGCGGCACAACCGGGACTCTCTCGGTTACGTGGTGGTGCTGGAGGTATGGGCTATCGTGCAGCAACCACTCAGGCCGAACAGCCAAGGTCAAGCCTATTGGACACCATTGGTCGGTTCGCTAAGGCTGGTGCCGATATGTATACCGCTAAGGAACAACGAGCACGAGACCTAGCTGATGAACGCTATAACGAGATTATCCGTAAGCTGACCCCTGA‑GCAACGTCGAGAAGCTCTCAACAACGGGACCCTTCTGTATCAGGATGACCCATACGCTATGGAAGCACTCCGAg                                                                                                                                                           <  1:238974/300‑1 (MQ=255)`  
                                                                                                                                                          `ttCAAGCGGCACAACCGGGACTCTCTCGGTTACGTGGTGGTGCGGGAGGTATGGGCTATCGTGCAGCAACCACTCAGGCCGAACAGCCAAGGTCAAGCCTATTGGACACCATTGGTCGGTTCGCTAAGGCTGGTGCCGATATGTATACCGCTAAGGAACAACGAGCACGAGACCTAGCTGATGAACGCTCTAACGAGATTATCCGTAAGCTGACCCCTGA‑GCAACGTCGAGAAGCTCTCAACAACGGGACCCTTCTGTATCAGGATGACCCATACGCTATGGAAGCACTCCGAGTCAAGAc                                                                                                                                                    <  1:482599/301‑1 (MQ=255)`  
                                                                                                                                                          `gtcggagatgtgtataagagacagctcGGTTACGTGGTGGTGCTGGAGGTATGGGCTATCGTGCAGCAACCACTCAGGCCGAACAGCCAAGGTCAAGCCTAGTGGACACCATTGGTCGGTTCGCTAAGGCTGGTGCCGATATGTATGCCGCTAAGGAACAACGAGCACGAGACCTAGCTGATGAACGCTCTAACGAGATTATCCGTAAGCTGACCCCTGA‑GCAACGTCGAGAAGCTCTCAACAACGGGACCCTTCTGTATCAGGATGACCCATACGCTATGGAAGCACTCCGAGTCAAGAc                                                                                                                                                    <  1:156274/277‑1 (MQ=255)`  
                                                                                                                                                           `tCAAGCGGCACAACCGGGACTCTCTCGGTTACGTGGTGGTGCTGGAGGTATGGGCTATCGTGCAGCAACCACTCAGGCCGAACAGCCAAGGTCAAGCCTATTGGACACCATTGGTCGGTTCGCTAAGGCTGGTGCCGATATGTATGCCGCTAAGGAACAACGAGCACGAGACCTAGCTGATGAACGCTCTAACGAGATTATCCGTAAGCTGACCCCTGA‑GCAACGTCGAGAAGCTCTCAACAACGGGACCCTTCTGTATCAGGATGACCCATACGCTATGGAAGTACTCCGAGTCAAGACt                                                                                                                                                   >  1:454103/1‑301 (MQ=255)`  
                                                                                                                                                                      `agccGGGACTCTCTCGGTTACGTGGGGGTGCTGGAGGTATGGGCTATCGTGCAGCAACCACTCAGGCCGAACAGCCAAGGTCAAGCCTATTGGACACCATTGGTCGGTTCGCTAAGGCTGGTGCCGATATGTATACCGCTAAGGAACAACGAGCACGAGACCTAGCTGATGAACGCTCTAACGAGATTATCCGTAAGCTGACCCCTGA‑GCAACGTCGAGAAGCTCTCAACAACGGGACCCTTCTGTATCAGGATGACCCATACGCTATGGAAGCACTCCGAGTCAAGACTGGTCGTAACGc                                                                                                                                        <  1:59357/299‑1 (MQ=255)`  
                                                                                                                                                                            `gtataagagacagaCGTGGTGGTGCTGGAGGTATGGGCTATCGTGCAGCAACCACTCAGGCCGAACAGCCAAGGTCAAGCCTATTGGACACCATTGGTCGGTTCGCTAAGGCTGGTGCCGATATGTATACCGCTAAGGAACAACGAGCACGAGACCTAGCTGATGAACGCTCTAACGAGATTATCCGTAAGCTGACCCCTGA‑GCAACGTCGAGAAGCTCTCAACAACGGGACCCTTCTGTATCAGGATGACCCATACGCTATGGAAGCACTCCGAGTCAAGACTGGTCGTAACGCTGCGTa                                                                                                                                  <  1:152159/288‑1 (MQ=255)`  
                                                                                                                                                                              `ctctctCGGTTACGTGGTGGTGCTGGAGGTATGGGCTATCGTGCAGCAACCACTCAGGCCGAACAGCCAAGGTCAAGCCTATTGGACACCATTGGTCGGTTCGCTAAGGCTGGTGCCGATATGTATGCCGCTAAGGAACAACGAGCACGAGACCTAGCTGATGAACGCGCTAACGAGATTATCCGTAAGCTGACCCCTGA‑GCAACGTCGAGAAGCTCTCAACAACGGGACCCTTCTGTATCAGCATGTCCCATACGCTATGACATCTCTCCTATTCAAGACTTGTCGTAACGCTGCGttat                                                                                                                                >  1:287969/1‑298 (MQ=255)`  
                                                                                                                                                                                 `tctcGGTTACGTGGTGGTGCTGGAGGTATGGGCTATCGTGCAGCAACCACTCAGGCCGAACAGCCAAGGTCAAGCCTATTGGACACCATTGGTCGGTTCGCTAAGGCTGGTGCCGATATGTATGCCGATAAGGAACAACGAGCACGAGACCTAGCTGATGAACTCTCTAACGAGATTATCCGTAAGCTGACCCCTGA‑GCAACGTCGAGAAGCTCTCAACAACGGGACCCTTCTGTATCAGGATGACCCATACGCTATGGAAGCACTCCGAGTCAAGACTGGTCGTAACGCTGCGTATCTtg                                                                                                                             <  1:229811/301‑1 (MQ=255)`  
                                                                                                                                                                                      `gTTACGTGGTGGTGCTGGAGGTATGGGCTATCGTGCAGCAACCACTCAGGCCGAACAGCCAAGGTCAAGCCTATTGGACACCATTGGTCGGTTCGCTAAGGCTGGTGCCGATATGTATACCGCTAAGGAACAACGAGCACGAGACCTTGATGATGAACGCTCTAACGGGAATATCCGTAAGCGCACCCCTGG‑GCAACGGCGAGAAGCTCTCAACAACGGGACCCTTCTGTATCAGGATGACCCATACGCGATGGAAGCAATCGGAGTCAAAACTGCGCGTACCGCTTGGTAACTGCTGGAc                                                                                                                        >  1:316610/1‑301 (MQ=255)`  
                                                                                                                                                                                      `gTTACGTGGTGGTGCTGGAGGTATGGGCTATCGTGCAGCAACCACTCAGGCCGAACAGCCAAGGTCAAGCCTATTGGACACCATTGGTCGGTTCGCTAAGGCTGGTGCCGATATGTATACCGCTAAGGAACAACGAGCACGAGACCTAGCTGATGAACGCTCTAACGAGATTATCCGTAAGCTGACCCCTGA‑GCAACGTCGAGAAGCTCTAAACAACGGGACCCTTCTGTATCAGGATGACCCCTACGCTATGGAAGCACTCCGAGTCAAGACTGGTCGTAACGCTGCGTAACTTGTGGag                                                                                                                        >  1:510619/1‑300 (MQ=255)`  
                                                                                                                                                                                                 `gtgCTGGAGGTATGGGCTATCGTGCAGCAACCACTCAGGCCGAACAGCCAAGGTCAAGCCTATTGGACACCATTGGTCGGTTCGCTAAGGCTGGTGCCGATATGTATACCGCTAAGGAACAACGAGCACGAGACCTAGCTGATGAACGCTCTAACGAGATTATCCGTAAGCTGACCCCTGA‑GCAACGTCGAGAAGCTCTCAACAACGGGACCCTTCTGTATCAGGATGACCCATACGCTATGGAAGCACTCCGAGTCAAGACTGGTCGTAACGATGCGTAGCTTGTGGACggtctcttata                                                                                                             >  1:35541/1‑291 (MQ=255)`  
                                                                                                                                                                                                   `gCTGGAGGGATGGGCTATCGTGAAGCAACCAGTCAGGCCGAACAGCCAAGGTTAAGCCTATTGGACACCATTGGGCGGTTCGCTAAGGATGGTGCCGATATGTATACCGCTAAGGAACAACGAGCACGAGACCTAGCTGATGAACGCTCTAACGAGATTATCCGTAAGCTGACCCCTTA‑GCAACGTCGAGAAGCTATCACAAACGGGACCCTTCTGTATCAGGATGACCCATACGCTATGGAAGCACTCCGAGTCAAGACTGGTCGTAACGCTGCGTATCTTGTGGACGATGACGTTATGc                                                                                                           <  1:41436/301‑1 (MQ=255)`  
                                                                                                                                                                                                   `ataagagacaggggCTATCGTGCAGCAACCACTCAGGCCGAACAGCCAAGGTCAAGCCTATTGGACACCATTGGTCGGTTCGCTAAGGCTGGTGCCGATATGTATGCCGCTAAGGAACAACGAGCACGAGACCTAGCTGATGAACGCTCTAACGAGATTATCCGTAAGCTGACCCCTGA‑GCAACGTCGAGAAGCTCTCAACAACGGGACCCTTCTGTATCAGGATGACCCATACGCTATGGAAGCACTCCGAGTCAAGACTGGTCGTAACGCTGCGTATCTTGTGGACGATGACGTTATGc                                                                                                           <  1:346071/290‑1 (MQ=255)`  
                                                                                                                                                                                                         `ggTATGGGCTATCGTGCAGCAACCACTCAGGCCGAACAGCCAAGGTCAAGCCTATTGGACACCATTGGTCGGTTCGCTAAGGCTGGTGCCGATATGTATGCCGCTAAGGAACAACGAGCACGAGACCTCGCTGATTAACGCTCTAACGAGATTATCCGTAAGCTGACCCCTGA‑GCAACGTCGAGAAGCTCTCAACAACGGGACCCTTCTGTATCAGGATGACTCATACGCTTTGGAAGCACTCCGAGTCAAGACTGGTCGTAACGCTGCGTATCTGTTGGACGATGACGTTATGCagcaga                                                                                                     >  1:2953/1‑301 (MQ=255)`  
                                                                                                                                                                                                         `ggTATGGGCTATCGTGCAGCAACCACTCAGGCCGAACAGCCAAGGTCAAGCCTATTGGACACCATTGGTCGGTTCGCTAAGGCTGGTGCCGATATGTATACCGCTAAGGAACCACGAGCACGAGACCTAGCTGATGAACGCTCTAACGAGATTATCCGTAAGCTGACCCCTGA‑GCAACGTCGAGAAGCTCTCAACAACGGGACCCTTCTGTATCAGGATGACCCATACGCTATGGAAGCACTCCGAGTCAAGACTGGTCGTAACGCTGCGTATCTTGTGGACGCTGCCGTTATGCagcaga                                                                                                     >  1:435218/1‑301 (MQ=255)`  
                                                                                                                                                                                                         `ggTATGGGCTATCGTGCAGCAACCACTCAGGCCGAACAGCCAAGGTCAAGCCTATTGGACACCATTGGTCGGTTCGCTAAGGCTGGTGCCGATATGTATACCGCTAAGGAACAACGAGCACGAGACCTAGCTGATGAACGCTCTAACGAGATTATCCGTAAGCTGACCCCTGA‑GCAACGTCGAGAAGCTCTCAACAACGGGACCCTTCTGTATCAGGATGACCCATACGCTATGGAAGCACTCCGAGTCAAGACTGGTCGTAACGCTGCGTATCTTGTGGACGATGACGtctgtctcttat                                                                                                     >  1:419344/1‑290 (MQ=255)`  
                                                                                                                                                                                                              `gggCTATCGTGCAGCAACCACTCAGGCCGAACAGCCAAGGTCAAGCCTATTGGACACCATTGGTCGGTTCGCTAAGGCTGGTGCCGATATGTATGCCGCTAAGGAACAACGAGCACGAGACCTAGCTGATGAACGCTCTAACGAGATTATCCGTAAGCTGACCCCTGA‑GCAACGTCTAGAAGCTCTCAACAACGGGACCCTTCTGTATCAGGATGACCAATACGCTATGGAAGCACTCCGAGTCAAGACTGGTCGTAACGCTGCGTATCTTGTGGAcctgtcccttatacacatctccgag                                                                                                >  1:486297/1‑277 (MQ=255)`  
                                                                                                                                                                                                               `ggCTATCGTGCAGCAACCACTCAGGCCGAACAGCCAAGGTCAAGCCTATTGGACACCATTGGTCGGTTCGCTAAGGCTGGTGCCGATATGTATACCGCTAAGGAACAACCAGCCCGAGACCTATCTGATGAACGCTCTATCGAGATTATCTGTAAGCTGACCCCTGA‑GCAACGTCGAGAAGCTCTCAACAACGGGACCCTTCTGTATAAGGATGACCCATACCCTAAGGAAGCACTCCGAGTCAAGACTGGTCGTAACGCTGCGTATCTTGTGGACGCTGACGTTATGCAGCAGaaaaaaa                                                                                               >  1:74159/1‑300 (MQ=255)`  
                                                                                                                                                                                                               `ggCTATCGTGCAGCAACCACTCAGGCCGAACAGACAAGGTAAAGCCTATTGGACACCATTGGTCGGTTCGCTAAGGCTGGTGCCGATATGTATGCCGCTAAGGAACAACGAGCACGAGACCTAGCTGATGAACGCTCTAACGAGATTATCCGTAAGCTGACCCCTGA‑GCAACGTCGAGAAGCTCTCAACAACGGGACCCTTCTGTATCAGGATGACCCATACGCTATGGAAGCACTCCGAGTCAAGACTGGTCGTAACGCTGCGTATCTTGTGGACGATGACGTTATGCAGCAGATAAAag                                                                                               <  1:310948/301‑1 (MQ=255)`  
                                                                                                                                                                                                               `gcggagatgtgtataagagacagggCCGAACATCCAAGGTCAAGCCTATTGGACACCATTGGTCGGTTCGCTAAGGCTGGTGCCGATATGTATACCGCTAAGGAACAACGAGAACGAGACCTAGCTGATGAACGCTCTAACGAGATTATCCGTAAGCTGACCCCTGA‑GCAAAGTCGAGAAGCTCTCAACAACGGGACCCTTCTGTATCAGGATGACCCATACGCTATGGAAGCACTCCGAGTCAAGACTGGTCGTAACGCTGCGTATCTTGTGGACGATGACGTTATGCAGCAGATAAAag                                                                                               <  1:213340/278‑1 (MQ=255)`  
                                                                                                                                                                                                                `aagaTCGTGCAGCAACCACTCAGGCCGAACAGCCAAGGTCAAGCCTAGTGGACACCATTGGTCGGTTCGCTAAGGCTGGTGCCGATATGTATACCGCTAAGGAACAACGAGCACGAGACCTAGCTGATGAACGCTCTAACGAGATTATCCGTAAGCTGACCCCGGA‑GCAACGTCGAGAAGCTCTCAACAACGGGACCCTTCTGTATCAGGATGACCCATACGCTATGGAAGCACTCCGAGTCAAGACTGGTCGTAACGCTGCGTATCTTGTGGACGATGACGTTATGCAGCAGATAAAaga                                                                                              <  1:234185/298‑1 (MQ=255)`  
                                                                                                                                                                                                                   `aTCGTGCAGCAACCACTCAGGCCTAACAGCCAAGGTCAAGCCTATTGGACACCATTGGTCGGTTCGCTAAGGCTGGTGCCGATATGTATGCCGCTAAGTAACAACGAGCACGAGACCTAGCTGATGAACGCTCTAACGAGATTATCCGTAAGCTGACCCCTGA‑GCAACGTCGAGAAGCTCTCAACCACGGGACCCTTCGTTATCAGGATGACCCATACGCTATGGAAGCACTCCGAGTCAAGACTGGTCGTAACGCTGCGTAACTTGTGGACGATGACGTTATGCAGAAGATAAAAGAggg                                                                                           >  1:16084/1‑301 (MQ=255)`  
                                                                                                                                                                                                                   `aTCGTGCAGCAACCACTCAGGCCGATCAGCCAAGGTCAAGCCTATTGGACACCATTGGTCGGTTCGCTAAGGCTGGTGCCGATATGTATGCCGCTAAGGAACAACGAGCACGAGACCTATCTGATGAACGCTCTAACGAGATTATCCGTAAGCTGACCCCTGA‑GCAACGTCGAGAAGCTCCCACCAACGGGACCCTTCTGTCTCTGGATGACCCATACGCTATGGAAGCACTCCGAGTCAAGACTGGTCTTAACGCTGCGTATCTTGTGGCCGATGACGTTATGCATCAGATAAAAGAtgg                                                                                           >  1:397422/1‑301 (MQ=255)`  
                                                                                                                                                                                                                   `aTCGTGCAGCAACCACTCAGGCCGAACAGCCAAGGTCAAGCCTATTGGACACCATTGGTCGGTTCGCTAAGGCTGGTGCCGATATGTATGCCGCTAAGGAACAACGAGCACGAGACCTAGCTGATGAACGCTCTAACGAGATTATCCGTAAGCTGACCCATGA‑GCAACGTCGAGAAGCTCTCAACAACGGGACCCTTCTGTATCAGGATGACCCATACGCTATGGAAGCACTCCGAGTCAAGACTGGTCGTAACGCTGCGTATCTTGTGGACGATTACGTTCTGCAGCAGATaaacgctgt                                                                                           >  1:91643/1‑295 (MQ=255)`  
                                                                                                                                                                                                                   `aTCGTGCAGCAACCACTCAGGCCGAACAGCCAAGGTCAAGCCTATTGGACACCATTGGTCGGTTCGCTAAGGCTGGTGCCGATATGTATACCGCTAAGGAACAACGAGCACGAGACCTAGCTGATGAACGCTCTAACGAGATTATCCGTAAGCTGACCCCTGA‑GCAACGTCGAGAAGCTCTCAACAACGGGACCCTTCTGTATCAGGATGACCCATACGCTATGGAAGCACTCCGAGTCCAGACTGGTCGTAACGCTGCGTATCTTGTGGACGATGACGTTATGCAGCAGATAAAAGAggg                                                                                           >  1:11604/1‑301 (MQ=255)`  
                                                                                                                                                                                                                      `gTGCAGCAACCACTCAGGCCGAACAGCCAAGGTCAAGCCTATTGGACACCATTGGTCGGTTCGCTAAGGCTGGTGCCGATATGTATACCGCTAAGGAACAACGAGCACGAGACCTAGCTGATGAACGCTCTAACGAGATTATCCGTAAGCTGACCCCTGA‑GCAACGTCGAGAAGCTCCCAACAACGGGACCCTTCTGTATCAGGATGACCCATACGCTATGGAAGCACTCCGAGTCAAGACTGGTCGTAATGCTGCGTATCTTGTGGACGATTACGTTATGCAGCAGATAAAagctgtctc                                                                                        >  1:222458/1‑294 (MQ=255)`  
                                                                                                                                                                                                                               `gagacagggCCGAACAGCCAAGGTCAAGCCTATTGGACACCATTGGTCGGTTCGCTAAGGCTGGTGCCGATATGTATGCCGCTAAGGAACAACGAGCACGAGACCTAGCTGATGAACGCTCTAACGAGATTATCCGTAAGCTGACCCCTGA‑GCAACGTCGAGAAGCTCTCAACAACGGGACCCTTCTGTATCAGGATGACCCATACGCTATGGAAGCACTCCGAGTCAAGACTGGTCGTAACGCTGCGTATCTTGTGGACGATGACGTTATGCAGCAGATAAAAGAGGGTGTCTTCCGTAc                                                                               <  1:243325/294‑1 (MQ=255)`  
                                                                                                                                                                                                                                      `ggCCGAACAGCCAAGGTCAAGCCTATTGGACACCATTGGTCGGTTCGCTAAGGCTGGTGCCGATATGTATGCCGCTAAGGAACAACGAGCACGAGACCTAGCTGATGAACGCTCTAACGAGATTATCCGTAAGCTGACCCCTGA‑GCAACGTCGAGAAGCTCTCAACAACGGGACCCTTCTGTATCAGGATGACCCATACGCTATGGAAGCACTCCGAGTCAAGACTGGTCGTAACGCTGCGTATCTTGTGGACGATGACGTTATGCAGCAGATAAAAGAGGGTGTCTTCCGTACTCGCGaa                                                                        >  1:217581/1‑301 (MQ=255)`  
                                                                                                                                                                                                                                             `cAGCCAAGGTCAAGCCTATTGGACACCATTGGTCGGTTCGCTAAGGCTGGTGCCGATATGTATGCCGCTAAGGAACAACGAGCTCGAGACCTAGCTGATGAACGCTCTAACGAGATTATCCGTAAGCTGACCCCTGA‑GCAACGTCGAGAAGCTCTCAACAACGGGACCCTTCTGTATCAGGATGACCCATACGCTATGGAAGCACTCCGAGTCAAGACTGGTCGTAACGCTGCGTATCTTGTGGACGATGACGTTATGCAGCAGATAAAAGAGGGTGTCTTCCGTACTCGCGAAGAGATgg                                                                 >  1:506747/1‑301 (MQ=255)`  
                                                                                                                                                                                                                                                  `gagacagaGCCTATTGGACACCATTGGTAGGTTCGCTAAGGCTGGTGCCGATATGTATACCGCTAAGGAACAACGAGCACGAGACCTAGCTGATGAACGCTATAACGAGATTATCCGTAAGCTGACCCCTGA‑GCAACGTCGAGAAGCTCTCAACAACGGGACCCTTCTGTATCAGGATGACCAATACGCTATGGAAGCACTCCGAGTCAAGACTGGTAGTAACGCTGCGTATCTTGTGGACGATGACGTTATGCAGCAGATAAAAGAGGGTGTCTTCCGTACTCGCGAAGAGATGGAagag                                                            <  1:185380/294‑1 (MQ=255)`  
                                                                                                                                                                                                                                                    `ggTCAAGCCTATTGGACACCATTGGTCGGTTCGCTAAGGCTGGTGCCGATATGTATGCCGCTAAGGAACAACGAGCACGAGACCTAGCTGATGAACGCTCTAACGAGATTATCCGTAAGCTGACCCCTGA‑GCAACGTCGAGAAGCTCTCAACAACGGGACCCTTCTGTATCAGGATGACCCATACGCTATGGAAGCACTCCGAGTCAAGACTGGTCGTAACGCTGCGTATCTTGTGGACGATGACGTTATGCAGCAGATAAAAGAGGGTGTCTTCCGTACTCGCGAAGAGATGGactgtct                                                          >  1:134343/1‑295 (MQ=255)`  
                                                                                                                                                                                                                                                     `gTCAAGCCTATTTTACACCATTGGTCGGTTCGCTAAGGCTGGTGCCGATATGTATACCGCTAAGGAACAACGAGCACGAGACCTAGCTGATGAACGCTCTAACGAGATTATCCGTAAGCTGACCCCTGA‑GCAACGTCGAGAAGCTCTCAACAACGGGACCCTTCTGTATCAGGATGACCCATACGCTATGGAAGCACTCCGAGTCAAGACTGGTCGTAACGCTGCGTATCTTGTGGACGATGACGTTATGCAGCAGATAAAAGAGGGTGTCTTCCGTACTCGCGAAGAGATGGAAGAGTAt                                                         <  1:100210/301‑1 (MQ=255)`  
                                                                                                                                                                                                                                                         `aGCCTATTGGACACCATTGGTCGGTTCGCTAAGGCTGGTGCCGATATGTATGCCGCTAAGGAACAACGAGCACGAGACCTAGCTGATGAACGCTCTAACGAGATTATCCGTAAGCTGACCCCTGA‑GCAACGTCGAGAAGCTCTCAACAACGGGACCCTTCTGTATCAGGATGACCCATACGCTATGGAAGCACTCCGAGTCAAGACTGGTCGTAACGCTGCGTATCTTGTGGACGATGACGTTATGCAGCAGATAAAAGAGGGTGTCTTCCGTACTCGCGAAGCGATGGAAGAGTATCggc                                                     >  1:405032/1‑299 (MQ=255)`  
                                                                                                                                                                                                                                                           `ccTATTGGACACCATTGGTCGGATCGATAAGGCTGGTGCCGATATGTATACCGCTAAGGAACAACGGGCACGAGACCTAGCTGATGAACGCTCTAACGAGATTATCCGTAAGCTGACCCCTGA‑GCAACGTCGAGAAGCTCTCAACAACGGGACCCTTCTGTATCAGGATGACCCATACGCTATGGAAGCACTCCGAGTCAAGACTGGTCGTAACGCTGCGTATCTTGTGGACGATGACGTTATGCAGCAGATAAAAGAGGGTGTCTTCCGTACTCGCGAAGAGATGGAAGAGTATAGCCAt                                                   <  1:321139/301‑1 (MQ=255)`  
                                                                                                                                                                                                                                                            `cTATTGGACACCATTGGTCGGTTCGCTAAGGCTGGTGCCGATATGTATGCCGCTAAGGAACAACGAGCACGAGACCTAGCTGATGAACGCTCTAACGAGATTATCCGTAAGCTGACCCCTGA‑GCAACGTCGAGAAGCTCTCAACAACGGGACCCTTCTGTATCAGGATGACCCATACGCTATGGAAGCACTCCGAGTCAAGACTGGTCGTAACGATGCGTATCTTGTGGACGATGAAGTTATGCAGCAGATAACAGAGGGTGTATTCCGTACTCGCGAAGAGATGGAAGAGTATCGCCATa                                                  >  1:338891/1‑301 (MQ=255)`  
                                                                                                                                                                                                                                                                  `gACACCATTGGTCGGTTCGCTAAGGCTGGTGCCGATATGTATGCCGCTAAGGAACAACGAGCACGAGACCTAGCTGATGAACGCTCTAACGAGATTATCCGTAAGCTGACCCCTGA‑GCAACGTCGAGAAGCTCTCAACAACGGGACCCGTCTGTATCAGGATGACCCATACGCTATGGAAGCACTCCGAGTCAAGACTGGTCGTAACGCTGCGTATCTTGTGGACTATGACGTTATGCAGCAGATAAAAGAGGGTGTCTTCCGTACTCGCGAAGAGATGGAAGAGTATCGCCATAGTCGcc                                            <  1:37536/301‑1 (MQ=255)`  
                                                                                                                                                                                                                                                                        `aTTGGTCGGTTCGCTAAGGCTGGTGCCGATATGTATACCGCTAAGGAACAACGAGCACGAGACCTAGCTGATGAACGCTCTAACGAGATTATCCGTAAGCTGACCCCTGA‑GCAACGTCGAGAAGCTATCAACAACGGGACCCTTCTGTATCAGGATGACCCATACGCTATGGAAGCACTCCGAGTCAAGACTGGTCGTAACGCTGCGTATCTTGTGGACGATGACGTTATGCAGCAGATAAAAGAGGGTGTCTTCCGTACTCGCGAAGAGATGGAAGAGTATCGCCATAGTCGCCTTCAag                                      <  1:500403/301‑1 (MQ=255)`  
                                                                                                                                                                                                                                                                         `ttGGTCGGTTCGCTAAGGCTGGTGCCGATATGGATACCGCTAAGGAACAACGAGCACGAGACCTAGCTGATGAACGCTCTAACGAGATTATCCGTAAGCTGACCCCTGA‑GCAACGTCGAGAAGCTCTCAACAACGGGACCCTTCTGTATCAGGATGACCCATACGCTATGGAAGCACTCCGAGTCAAGACTGGTCGTAACGCTGCGTATCTTGTGGACGATGACGTTATGCAGCAGATAAAAGAGGGTGTCTTCCGTACTCGCGAAGAGATGGAAGAGTATCGCCATAGTCGCCTTCAaga                                     <  1:274802/301‑1 (MQ=255)`  
                                                                                                                                                                                                                                                                          `gctcggagatgtgtataagagacaggATATGTATACCGCTAAGGAACAACGAGCACGAGACCTAGCTGATGAACGCTCTAACGAGATTATCCGTAAGCTGACCCCTGA‑GCAACGTCGAGAAGCTCTCAACAACGGTACCCTTCTGTATCAGGATTACCCATACGCTATGGAAGCACTCCGAGTCAAGACTGGTCGTAACGCTGCGTATCTTGTGGACGATGACGTTATGCAGCAGATAAAAGAGGGTGTCTTCCGTACTCGCGAAGAGATGGAAGAGTATCGCCATAGTCGCCTTCAagag                                    <  1:24905/276‑1 (MQ=255)`  
                                                                                                                                                                                                                                                                          `gagaaagttacgtAAGGGCGGTGCCGAAATGTAAGCCGGTAAAGAAACACGAGAAAGAGACCTTAAGGGAGAACGGTCTAACGGGAGTAACCGTAAGGCGACACCTGAGGCAAGGT‑GAAAAAGTCTCAAACACGGGACTCTTCTGTATCAGGATGACCCATACGCTATGGAAGCACTCCGAGTCAATACTGGTCGTAACGCTGCGTATCTTGTGGACGATGACGTTATGCAGCAGATAAAAGAGGGTGTCTTCCGTACTCGCGAAGAGATGGAAGAGTATCGCCATAGTCGCATTCAagag                                    <  1:279368/289‑1 (MQ=255)`  
                                                                                                                                                                                                                                                                                  `tCGCTAATGCCGGTGCCGATATGTATACCGATAAGGAACAGCTAGCACGACACCTAGCTGATGAACGCTCTACCTGGATTATCCGTAATCTGACCCCTGA‑GCAACGTCGAGCAGCTCTCAACAACGGGACCCTTCTGTATCAGGATGACCCATACGCTATGGAAGCACTCCGAGTAAAGACTGGTCGTAACGCTGCGTATCTTGTGGACGATGACGTTATGCAGCATATAAAAGAGGGTGTCTTCCGTACTCGCGAAGAGATGGAAGAGTATCGCCATAGTCGCCTTCAAGAGGGCGCTaa                            <  1:95631/301‑1 (MQ=255)`  
                                                                                                                                                                                                                                                                                   `cGCTAAGGCTGGTGCCGATATGTATACCGCTAAGGAACAACGAGCACGAGACCTAGCTGATGAACGCTCTAACGAGATTATCCGTAAGCTGACCCCTGA‑GCAACGTCGAGAAGCTCTCAACAACGGGACCCTTCTGTATCAGGATGACCCATACGCTATGGAAGCACTCCGAGTCAAGACTGGTCGTAACGCTGCGTATCTTGTGGACGATGACGTTATGCAGCAGATAAAAGAGGGTGTCTTCCGTACTCGCGAAGAGATGGAAGAGTATCGCCCTAGTCGCCTTCAAGAGGGCGCTAAg                           >  1:379708/1‑301 (MQ=255)`  
                                                                                                                                                                                                                                                                                    `gCTAAGGCTGGTGCCGATATGTATGCCGCTAAGGAACAACGAGCACGAGACCTAGCTGATGAACGCTCTAACGAGATTATCCGTAAGCTGACCCCTGA‑GCAACGTCGAGAAGCTCTCAACAACGGGACCCTTCTGTATCAGGATGACCCATACGCTATGGAAGCACTCCGAGTCAAGACTGGTCGTAACGCTGCGTATCTTGTGGACGATGACGTTATGCAGCAGATAAAAGAGGGTGTCTTCCGTACTCGCGAAGAGATGGAAGAGTATCGCCATAGTCGCCTTCAAGAGGGCGCTAAgg                          <  1:472383/301‑1 (MQ=255)`  
                                                                                                                                                                                                                                                                                         `ggCTGGTGCCGATATGTATACCGCTAAGGAACAACGAGCACGAGAGCTAGCTGATGAACGATCTAACGAGATTATCCGTAAGCTGACCCCTGA‑GCAACGTCGAGAAGCTCTCAACAACGGGACCCTTCTGTATCAGGATGACCCATACGCTATGGAAGCACTCCGAGTCAAGACTGGTCGTAACGCTGCGTATCGTGTGGACGATGACGTTATGCAGCAGATAAAAGAGGGTGTCTTCCGTACTCGCGAAGAGATGGAAGAGTATCGCCATAGTCGCCTTCAAGAGGGCGCTAAGGTATAc                     <  1:226010/301‑1 (MQ=255)`  
                                                                                                                                                                                                                                                                                         `gagacagcccGATATGGATGCCGCTAAGGAACAACGATCACGAGACCTAGCTGATGAACGCTCTAACGAGATTATCCGTAAGCTGACCCCTGA‑GCAACGTCGAGAAGCTCTCAACAACGGGACCCTTCTGTATCAGGATGACCCATACGCTATGGAAGCACTCCGAGTCAAGACTGGTCGTAACGCTGCGTATCTTGTGGACGATGACGTTATGCAGCAGATAAAAGAGGGTGTCTTCCGTACTCGCGAAGAGATGGAAGAGTATCGCCATAGTCGCCTTCAAGAGGGCGCTAAGGTATAc                     <  1:467000/293‑1 (MQ=255)`  
                                                                                                                                                                                                                                                                                           `ctgggtccGATATGTAAACCGATAAGGAACAACGAGAACGAGACCTAGCTGATGAACGCTCGAACGAGATTATCCGTAAGCTGACCCCTGA‑GCAACGTCGAGAAGCTCTCAACAACGGGACCCTTCTGTATCAGGATGACCCATACGCTATGGAAGCACTCCGAGTCAAGACTGGTCGTAACGCTGCGTATCTTGTGGACGATGACGTTATACAGCAGATAAAAGAGGGTGTCTTCCGTACTCGCGAAGAGATGGAAGAGTATCGCCATAGTCGCCTTCAAGAGGGCGCTAAGGTATACGc                   <  1:341768/295‑1 (MQ=255)`  
                                                                                                                                                                                                                                                                                           `cTGGTGCCGATATGTATACCGCTAAGGAACAACGAGCACGAGACCTAGCTGATGAACGCTCTAACGAGATTATCCGTAAGCTGACCCCTGA‑GCAACGTCGAGAAGCTCTCAACAACGGGACCCTTCTGTATCAGGATGACCCATACGCTATGGAAGCACTCCGAGTCAAGACTGGTCGTAACGCTGCGTATCTTGTGGACGATGACGTTATGCAGCAGATAAAAGAGGGTGTCTTCCGTACTCGCGAAGAGATGGAAGAGTATCGCCATAGTCGCCTTCAAGAGGGCGCTAAGGTATACGc                   <  1:189899/301‑1 (MQ=255)`  
                                                                                                                                                                                                                                                                                           `agagacaggATATGTATACCGCTAAGGAACAACGAGCACGAGACCTAGCTGATGAACGCTCTAACGAGATTATCCGTAAGCTGACCCCTGA‑GCAACGTCGATAAGCTCTCAACAACGGGACCCTTCTGTATCAGGATGACCCATACGCTATGGAAGCACTCCGAGTCAAGACTGGTCGTAACGCTGCGTATCTTGTGGACGATGACGTTATGCAGCAGATAAAAGAGGGTGTCTTCCGTACTCGCGAAGAGATGGAAGAGTATCGCCATAGTCGCCTTCAAGAGGGCGCTAAGGTATACGc                   <  1:110259/293‑1 (MQ=255)`  
                                                                                                                                                                                                                                                                                           `agacagCCGATATGTATGCCGCTAAGGAACAACGAGCACGAGACCTAGCTGATGAACGCTCTAACGAGATTATCCGTAAGCTGACCCCTGA‑GCAACGTCGAGAAGCTCTCAACAACGGGACCCTTCTGTATCAGGATGACCCATACGCTATGGAAGCACTCCGAGTCAAGACTGGTCGTAACGCTGCGTATCTTGTGGACGATGACGTTATGCAGCAGATAAAAGAGGGTGTCTTCCGTACTCGCGAAGAGATGGAAGAGTATCGCCATAGTCGCCTTCAAGAGGGCGCTAAGGTATACGc                   <  1:299980/296‑1 (MQ=255)`  
                                                                                                                                                                                                                                                                                                   `gATATGTATACCGCTAAGGAACAACGAGCACGAGACCTAGCTGATGAACTCTCTAACGAGATTATCCGTAAGCTGACCCCTGA‑GCAACGTCGAGAAGCTCTCAACAACGGGACCCTTCTGTATCAGGATGACCCAAACGCTATGGAAGCACTCCGAGTCAAGACTGGTCATAACGCTGCGTATCTTGTGGACGATGACGTTATGCAGCAGATAAAAGAGGGTGTCTTCCGTACTCCCGAAGTGATGGAAGAGTATCGCCCTAGTCGCCTTCAAGCGGGCGCTAAGGTATAcctgtctctta           >  1:196284/1‑291 (MQ=255)`  
                                                                                                                                                                                                                                                                                                     `tatgtatgCCGCTAAGGAACAACGAGCACGAGACCTAGCTGATGAACGCTCTAACGAGATTATCCGTAAGCTGACCCCTGA‑GCAACGTCGAGAAGCTCTCAACAACGGGACCCTTCTGTATCAGGATGACCCATACGCTATGGAAGCACTCCGAGTCAAGACTGGTCGTAACGCTGCGTATCTTGTGGACGATGACGTTATGCAGCAGATAAAAGAGGGTGTATTCCGTACTCGCGAAGAGATGGAAGAGTATCACCATAGTAGCCTTCAAGAGGGCGCTAAGGTATACGCTGAGCAGTTc         >  1:495765/1‑301 (MQ=255)`  
                                                                                                                                                                                                                                                                                                     `tatgtatgCCGCTAAGGAACAACGAGCACGAGACCTAGCTGATGAACGCTCTAACGAGATTATCCGTAAGCTGA‑CCCTGA‑GCAACGTCGAGAAGCTCTCAATAACGGGACCCTTCTGTATCAGGATGACCCATACGATATGGAAGCACTCCGAGTCAAGACTGGTCGTAACGCTGCGTATCTTGTGGACGATGACGTTATGCAGCAGATAAAAGAGGGTGTCTTCCGTACTCGCGAAGAGATGGAAGAGTATCGCCATAGCCGCCTTCAAGAGGCCGCTAAGGACTACGCTGAGCAGTTCg        >  1:78574/1‑301 (MQ=255)`  
                                                                                                                                                                                                                                                                                                        `gTATACCGCTAAGGAACAACGAGCACGAGACCTAGCTGATGAACGCTCTAACGAGATTATCCGTAAGCTGACCCCTGA‑GCAACGTCGAGAAGCTCTCAACAACGGGACCCTTCTGTATCAGGATGACCCATACGCTATGGAAGCACTCCGAGTCAAGACTGGTCGTAACGCTGCGTATCTTGTGGACGATGACGTTATGCAGCAGATAAAAGAGGGTGTCTTCCGTACTCGCGAAGAGATGGAAGAGTATCGCCATAGTCGCCTTCAAGCGGGCGCTAAGGTTTACGCTGAGCAGTTCgtc      >  1:351424/1‑299 (MQ=255)`  
                                                                                                                                                                                                                                                                                                          `aaaCCGCTAAGAAACAACGAACACGAGACCTATCTCATGATCGCGATAACGAGTTTAGCCATAATCTGACCCCAAA‑GCAACATCGAGAAGCACTCAACAACGGGACCCTTCTGTATCAGGAGAACCCAGACGCTAGTGAAGCACGCCGAGTCAAGACTAGTCATAACGATGCGTATTTTGTAGACGATGTCGTTATGCAGCAGATAAAAGAGGTTGTCTTCCGTACTCGCGAAGATATGGAAGAGTATCGCCATAGTCGCCTTCAAGAGGGCGATAAGGTATACGCTGAGCAGTTCGGCAt    <  1:334519/299‑1 (MQ=255)`  

                                                                                                                                                                                                                                                                                                            `|`                                                                                                                                                                                                                                                                                                               
`GTTCTGGACCCACTGGCTATCATGGGGTCTTCCGCTGCGAGTGCTTACGCATCCGGTGCGTTCGACTCTAAGTCCACAACTAAGGCACCTATTGTTGCCGCTAAAGGAACCAAGACGGGGAGGTAATGAGCTATGAGTAAAATTGAATCTGCCCTTCAAGCGGCACAACCGGGACTCTCTCGGTTACGTGGTGGTGCTGGAGGTATGGGCTATCGTGCAGCAACCACTCAGGCCGAACAGCCAAGGTCAAGCCTATTGGACACCATTGGTCGGTTCGCTAAGGCTGGTGCCGATATGTATACCGCTAAGGAACAACGAGCACGAGACCTAGCTGATGAACGCTCTAACGAGATTATCCGTAAGCTGACCCCTGA‑GCAACGTCGAGAAGCTCTCAACAACGGGACCCTTCTGTATCAGGATGACCCATACGCTATGGAAGCACTCCGAGTCAAGACTGGTCGTAACGCTGCGTATCTTGTGGACGATGACGTTATGCAGAAGATAAAAGAGGGTGTCTTCCGTACTCGCGAAGAGATGGAAGAGTATCGCCATAGTCGCCTTCAAGAGGGCGCTAAGGTATACGCTGAGCAGTTCGGCATCG  >  NC_001604.1/28193‑28793`

  

Alignment Legend

Aligned base mismatch/match (shaded by quality score): `ATCG/ATCG` `< 3 ≤` `ATCG/ATCG` `< 9 ≤` `ATCG/ATCG` `< 18 ≤` `ATCG/ATCG` `< 33 ≤` `ATCG/ATCG` `< 38 ≤` `ATCG/ATCG`

Unaligned base: `atcg`    Masked matching base: `atcg`    Alignment gap: `‑`    Deleted base: `‑`
